# A Permissible Sin: Perceptions of Smoking Among Haredi Men in Israel

**DOI:** 10.1007/s10943-024-02019-2

**Published:** 2024-03-15

**Authors:** Shlomo Guzmen-Carmeli, Rotem Weizman, Tammar Friedman

**Affiliations:** Department of Sociology and Anthropology, Bar Ilan University, 590002 Ramat Gan, Israel

**Keywords:** Permissible sin, Haredi, Health risks, Smoking

## Abstract

This essay deals with perceptions of smoking among Haredi men in Israel. Though trends in smoking within the Haredi society have been quantitively examined, no qualitative research has ever focused on the motivations and mindsets stimulating individuals’ choices to take health risks despite religious precepts to the contrary. Israeli Haredi men sometimes start smoking in their early childhood and are unmotivated to quit, and such circumstances should be examined. We interviewed 20 Israeli Haredi male smokers and overviewed the Haredi daily press and rabbinical attitudes toward smoking. Our findings indicate that Haredi men typically consider smoking as either permissible or, at worst, a minor sin. From childhood they view smoking as an expression of maturity, and moreover one which is associated with Jewish holidays and particular religious practices. Such perception relies on the Haredi establishment's normative exclusion of smoking from the Halachic commandments that aim to protect health. Finally, we illustrate key points to consider in paths leading to an intervention process to change these norms and practices.

## Introduction

*Smoking hazards*: Smoking cigarettes is a particularly high-risk behavior due to its high frequency and mortal danger (Rogers et al., 2005). In the Western world, raising awareness of the dangers of smoking and efforts to eradicate this phenomenon are part of institutional public and private health activities designed to improve national health. Notwithstanding, the scholarship identifies the possibility that stigmatizing smokers might offend socially less privileged groups and generate a sort of "desperate logic," i.e., creating a situation that makes eradicating smoking among these subaltern groups extremely difficult (Thompson et al., 2007).

*Smoking habit in Israel*: The rate of smokers in Israel is relatively high compared to the average rate among OECD countries. In 2020, the rate of smokers among adults in Israel was about 20%, with men smoking more than women. An Israeli Ministry of Health report indicates a stagnated rate of smoking, yet it identifies an increase in smoking in comparison with 2016 (IMH, 2021). However, the rate of Haredi smokers was lower than the rest of the population (ICBS, 2018). Significant transgressive behavioral aspects in this sector relate to its members' habitual smoking since early childhood and their lack of motivation to quit (Kopel et al., 2013).

*Efforts and obstacles in eradicating smoking*: The Israeli Ministry of Health actively works to reduce smoking and eradicate it through legislative acts, enforcement, taxation, and subsidized nicotine rehabilitation, and it recently began recommending increasing the active protection of the public from passive smoking (IMH, 2021, 2023). However, this raises an inquiry concerning the extent of institutional influence over the members of the Haredi group and their understanding of what constitutes health-risk behavior. As for the latter, for example, Arbel et al. (2023) identified a high rate of sugar consumption and obesity within the Haredi group. With regard to smoking habits, Kopel et al. (2013) suggest that smoking is not strictly prohibited in the Haredi society, as such prohibition is commonly regarded as a decree that the public cannot abide by. Their study also indicates that Haredi men are not influenced by some of the rabbinic leaders who have encouraged their congregations to quit smoking. A few respondents expressed that even if their rabbi quit smoking, they do not intend to follow him (Kopel et al., 2013).

*Toward intervention model*: Anshel (2010) presents the Disconnected Values Model (DVM) of health intervention to be held by the religious leadership, which does not require extensive professional training; but merely to recognize the negativity of this high-risk habit alongside presenting it as disconnected from main traditional values and contrasts smoking to Judeo-Christian scripts which praise habits that promote good health (Anshel, 2010). In this research, we focus on the phenomenon of smoking among the Haredi male population while trying to explicate the contradiction between the smokers' lifestyles and the biblical command "Watch yourselves very carefully" (Deuteronomy, 4, 15). Finally, we illustrate paths leading to an intervention process that might be useful in developing intervention plans for smoking cessation within the Haredi population.

## Literature Review

*Haredi sector in Israel*: Our research deals with trends in smoking among Haredi men who live in Israel and the crucial role of the leadership of this community in forming programs for intervention in Haredi men's lifestyle. In this context, the term Haredi refers to numerous religious groups in Jewish Orthodoxy, which researchers often consider as Haredi. However, this is a heterogenic sector that includes different ethnic groups, i.e., Haredim of Ashkenazi (Jewish East European) origins and Sephardic (Jewish Mediterranean, Middle Eastern, Asian, and African) origins, and diverse religious groups that strictly follow the rules of the Jewish Halacha (an ancient codex of Jewish religious laws) and share conservatism and various levels of separatism. Though Haredi groups initially rejected the Zionist movement, nowadays, most do not oppose the Zionist idea and strive toward mutual goals within the State of Israel's political system, while a small segment of the Haredi society attempts to deny the authority of the state and its institutions completely (see Heilman & Friedman, 1991; Leon, 2020; Malach & Cahaner, 2021; Taragin-Zeller & Stadler, 2022; Guzmen-Carmeli, 2020b) . Thus, for example, the Haredi community recruits one of the touchstones of liberal democracy to challenge Israel's prominent secular cultural values (Guzmen-Carmeli, 2013).

The Haredi group is a minority^1^ at the margins of the polarized, conflicted texture of Israeli society, and Leon (2020) explains that they are stigmatized and discriminated against as margins of Jewish sovereignty. Yet it is impossible to ignore their sovereign power in regard to their regulated Jewish lifestyle. The Haredi culture paradoxically constructs its identity based on its religious and ideological leadership on the one hand and this public's daily distress and marginal experiences on the other hand. This population's encounters with the health authorities, in particular, require a careful cultural adjustment (Flannelly et al. 2006; Taragin-Zeller & Stadler, 2022; Taragin-Zeller et al., 2022). Notably, 2019 research indicates the Haredi society's trust in the health system and the doctors; nevertheless, due to the particular structure of this sector, it relies on its own community system: for instance, incorporating rabbinic advice, separate economic organizations, or applying for existent private medicine, in accordance with rabbinic orders (Yuval et al., 2019).

*Smoking habit in the Haredi sector*: Pinchas-Mizrachi and Finkelstein (2022) indicate low smoking preclearance among ultra-orthodox men aged 50 years or older. However, the smoking phenomenon within the young Haredi population is understudied. For example, the Israeli Ministry of Health indicates that the average starting age for Haredi men is 17, and they initially experimented during early youth (IMH, 2011). Later, an IMH report from 2015 indicated a low rate of Haredi men starting to smoke between 14 and 17. Each year, the Israel Minister of Health submits an extensive report to the Israeli parliament about smoking in Israel—yet the report does relate to levels of religiosity. For example, the recent report relating to 2022 focuses on age groups, a specific chapter designated to smoking in the military service, sectoral differences between the Jewish and Arab populations, and extensive details relating to legislative acts and enforcement (IMH, 2023).

Haredi press indicates that the first cigarette is usually associated with a particular Jewish celebratory holiday, Purim. Smoking a cigarette on Purim accords with the popular custom of *venahafoch hu*, which means something along the lines of "turning everything upside down," purposely acting in complete opposition to social acceptability or appropriateness (Adamker, 2016; Farkash, 2013; Heller, 2011; Sela, 2008). Waitzman (2017) suggests that Haredi men starting to smoke at a young age is an act of rebellion; thus, efforts to irradicate smoking should begin at a young age.

Furthermore, the Haredi population's lack of motivation to stop smoking is conspicuous. Examination of the reasons for smoking, whether it derives from habit or addiction, reveals that most Haredim are simply not interested in quitting smoking even when their rabbis resist the practice (IMH, 2011). The scholarship deals with diverse reasons for high-risk behavior, whether such a behavior is unaware or relies on misperception and lack of knowledge about the health risks (Cummings et al., 2004); or excitement and emotional relief (Koren & Bony-Noach, 2011); or the misbelief that smoking does not harm one's health (Petek et al., 2006).

*What lies between smoking as a high-risk behavior and religiosity?* In their research of high-risk behaviors, Koren and Bony-Noach (2011) consider smoking as deviant yet voluntary behavior that negatively affects one's health and has social implications. Goode (2019) distinguishes between deviance on a normative level and deviance on a reactive level. Normatively, deviance is any transgression of a particular social norm; thus, deviant behavior should be examined in a particular societal context. Deviance on a reactive level relies on the other's point of view and is regarded as such only if the deviant behavior is condemned. Hence, what is at stake is not the act itself but the social response (Goode, 2019). Therefore, it is important to examine the meaning of the perception of smoking as deviant, and whether it is perceived as such, both normatively and reactively.

Normatively, the perception of smoking has sociocultural dimensions. American research suggests that affiliation with a particular faith denomination affects smoking frequency (Wasserman & Trovato, 1996). Additionally, there are differences in smoking habits between different religions, for example, in Britain Muslims smoke less than Christians (Hussain et al., 2019).

There are sociocultural dimensions of smoking that do not relate to religiosity, such as the connection between smoking and mental health for American female veterans (Wilson et al., 2019). As the Haredi way of life is structured and controlled by strict interpretations of the Jewish laws of halacha (Brown, 2017), the Haredi man's daily routine is full of commandments (*Mitzvot*) and customs, whereby he is expected to adjust his behavior and conform in accordance with his community's norms. Thus, we inquired: Is smoking perceived as assisting in reducing stress? How is smoking integrated into the Haredi way of life? The latter relates to our desire to examine whether there is a connection between religious lifestyle and a choice to smoke.

Brammli-Greenberg et al. (2018) indicate there is no connection between the religiosity level and the health of Israeli Jews. Individuals who identified as haredi and individuals who identified as secular were healthier than the population centered on the axis. Regarding smoking, Brown et al. (2014) suggest no correlation between participating in religious activities and smoking. On the other hand, Yong et al. (2013) indicate differences between religious and secular groups. As opposed to the latter, Muslims and Buddhists in Southeast Asia recognize the significant positive influence of moral values on the smoking rate (Yong et al., 2013). Seemingly, religious organizations can be powerful in advancing health issues among their members (Schoenberg, 2017).

*Actions to eradicate lifestyle and the cultural dimension*: Rabbi Chaim Landau, Tel Aviv's Chief Rabbi in the early 1970s, strictly forbade smoking, stating to the press that smoking is not in accordance with halacha. Other rabbis, however, did not agree with his position on this matter. Many rabbis are smokers, and in their eyes, the risk of smoking refers to the future, not the present; thus, quitting smoking is not obligatory. According to Rabbi Landau, forbidding smoking is based on Maimonides' thoughts and the halachic law that forbids self-harming behaviors. In contrast, many rabbis believe that the damage of smoking is merely a possibility; as such, they do not forbid it (Benson, 1994). Added to that, in their study, Knishkowy et al. (2012) investigated the effect of rabbinic proclamations regarding tobacco use and smoking habits on haredi adolescents. The researchers found that the leaflets intended to eradicate this phenomenon failed to demonstrate the impact on adolescent haredi smokers.

Smoking is perceived as a response to the tensions built into the demanding lifestyle of the Haredi man, which may perhaps help its perception as a minor offense that does not overrule halachic laws. Yet it is noteworthy that the scholarship is ambivalent concerning the apparent advantage of smoking as a means of dissipating anxiety, and it stresses that smoking mainly increases anxiety (Silverstein, 1982; Kassel et al., 2003). Considering the typically strict halachic culture of haredi groups (Samet, 2005), smoking can perhaps best be described as a "permissible sin." In other words, it is a minor social norm violation that can even be perceived as an acceptable means of releasing cultural stress and preserving and maintaining conformity and social control among this public (Friedman, 1991). Hence, even when the Haredi man is aware of his risky behavior, he might create a system justification that rationalizes his smoking choice. This coincides with research about the power of self-conviction; in attempting to achieve a certain goal, one can create a justification system that validates one’s choice, even retrospectively (Winchester & Green, 2019).

Historically, religion significantly affected the development of medical establishments' ethics, yet it did not affect preventive medicine. The latter can be influenced by experts' knowledge and through Rabbinic advice. Notably, traditional religious perspectives and modern perceptions of social justice and public health can be enlisted for this purpose (Rozier, 2017). Acknowledging the cultural dimension of smoking norms within this community is essential. Not condemning smokers within Haredi society and perceiving this habit as health-risky but non-deviant counteracts the possibility of perceiving smoking as reactive deviation. Research into Haredim in Israel indicates their yearning for smoking and consequent anxiety are conspicuously lower on Saturdays than on weekdays (Dar et al., 2005); thus, in terms of deviation from normativity, a strict halachic prohibition against smoking could greatly influence smoking habits in the Haredi population, especially given that research indicates the significance of rabbinic intervention in promoting a congregation's health (Flannelly et al. 2006).

## Methodology

Assuming qualitative research is a useful tool to address the topic of Haredi men's choice to smoke and their interpretations, while exploring its explicit and implicit dimensions, we used semi-structured interviews (Babbie, 2020) and textual content analysis (Atkinson & Coffey, 2004) as research tools. This paper is part of extensive research about the Haredi society and pertains to responses relating to the respondents' smoking habits and perspectives. The participants were recruited via snowball sampling. The first author has conducted significant field research in the Haredi community over the past decade (Sharabi & Guzmen-Carmeli, 2013; Guzmen-Carmeli & Rubin, 2014; Guzmen-Carmeli & Sharabi, 2014, 2019; Guzmen-Carmeli, 2020a, b; Fuchs et al. 2024). The second author conducts a study among Haredi adults and young teenagers. Given their access to this sector, they could approach individuals who smoke among different haredi groupings. Familiarity with the study participants facilitated the establishment of the essential trust required to execute our research. Interviews conducted in 2018 were paused during the Covid-19 outbreak and continued until 2021. Though many researchers engaged in Zoom and telephone interviews during the COVID-19 pandemic outbreak, such a method was not appropriate for our research participants.

We conducted 20 interviews with Haredi male smokers; all graduated from the Haredi education system. As Haredi society is diverse, we addressed men from various Haredi groups: Lithuanian Haredim and Hassidim, and Ashkenazi and Sephardic Haredim (see Table 1, participants demographic). We interviewed participants who were born in Haredi society and not Baali Tshuva (repentant) in order to avoid premises that their smoking habits could possibly relate to their former secular background. The high age range of respondents (21–62) is intentional—We aim to demonstrate that smoking practices begin at a young age and last for a long time. The choice in the broad age range of respondents aims to emphasize the reasoning and meanings of smoking as a prevalent phenomenon in various age groups.

**Table 1:** Participants demographic characteristics

Pseudo name	Age	Residence	Haredi group	Interview held
Benjamin	62	Jerusalem	Lithuanian Haredi	2020
Eli	55	Jerusalem	Sephardic Haredi	2021
Itzhak	50	Modi'in Ilit	Hasidic	2019
David	50	Jerusalem	Sephardic Haredi	2020
Sholem	49	Modi'in Ilit	Lithuanian Haredi	2019
Menashe	46	Modi'in Ilit	Lithuanian Haredi	2019
Joel	38	Modi'in Ilit	Lithuanian Haredi	2019
Eithan	38	Modi'in Ilit	Lithuanian Haredi	2019
Jacob	36	Modi'in Ilit	Hasidic	2019
Mendel	36	Beitar Ilit	Lithuanian Haredi	2020
Shimon	33	Modi'in Ilit	Lithuanian Haredi	2019
Pinhas	31	Bene Brak	Sephardic Haredi	2019
Abraham	30	Beit Shemesh	Hasidic	2021
Evyatar	29	Bene Brak	Sephardic Haredi	2019
Asher	28	Jerusalem	Hasidic	2020
Yosef	28	Tiberias	Sephardic Haredi	2020
Harel	26	Modi'in Ilit	Lithuanian Haredi	2019
Eliyahu	24	Beit Shemesh	Sephardic Haredi	2021
Rachamim	23	Modi'in Ilit	Hasidic	2019
Reuven	21	Jerusalem	Hasidic	2020

We preferred semi-structured interviews, which enable flexibility in responding. Part of the questionnaire integrates conventional questionnaires from Israeli health services organizations that indicate the interviewee's level of nicotine dependence. The questionnaire aims to collect relevant information, alongside integrating inquiries relating to the interviewees' perception of halachic and rabbinic attitudes toward smoking. The first and second writers conducted the interviews. The interviews lasted between 30 min to 1 h and were conducted face-to-face, recorded, and transcribed.

Regarding ethical issues, researching the Haredi population and signing a written document of an informed consent form may create fear and a lack of cooperation. Therefore, to obtain informed consent from all the interviewees, consent was given orally and recorded at the beginning of the interviews after the purpose of the study was explained to them, and they were assured that the anonymity of the interviewees would be preserved. The Bar-Ilan University Ethics Committee approved the study.

In our content analysis, we referred to halachic literature and media coverage of current trends in smoking within Haredi society. We have thematically reviewed terms such as smoking, cigarette, and the use of tobacco in the following Haredi press online archives: *Kikar Ha-shabat, Behadrei Hadarim, He Mishpacha, and Arutz Sheva*, which is not a Haredi website but has many writers and readers from the Haredi sector. To understand the rabbinical position regarding smoking and tobacco use, we reviewed Haredi responsa literature from the past decades (1980–2021). In addition, we turned to rabbis and halachic Haredi figures and asked for references to sources dealing with the subject. Similar to what appeared in Weizman's article (2017), we were surprised by the paucity of engagement with this topic in the Haredi press and the rabbinic responsa literature.

We introduced the interviewees to these publications and asked their opinions about them. For example, the interviewees spoke about a 2008 campaign by the Mayor of Jerusalem to reduce smoking among Haredim. Notably, this campaign did not include the influential perspective of relevant rabbis. Data collected were then thematically analyzed according to a model from Miles et al. (2018), which provides for initial interpretative reading, reducing, and conceptualizing the material and its thematic processing. All three writers engaged with analysis. In analyzing the interviews, we indicated the main themes uncovered relating to smoking among men in Haredi society.

In efforts to maintain rich rigor, according to Tracy (2010: 841), we ensured: (a) our data support our significant claims; (b) a sufficient amount of time was spent to gather information; (c) The context aligns with the objectives of our study since qualitative research seeks to elucidate a phenomenon and capture the perspectives of the participants.

### Findings

The interviews indicate common characteristics of Haredi men's choosing to smoke, including their consideration of smoking as a coming of age ceremony (rite of passage) performed since early childhood, common perception of health risks, the biblical commandment "And you shall safeguard your soul" (Deuteronomy, 4, 15); and the influence of the Haredi leadership that regularly avoids any action against smoking.

### Smoking as a Coming of age Ceremony (Rite of Passage)

Most interviewers indicated that they started smoking when they had a chance to masquerade as adults on Purim, in which the custom of *va'nahafoch hoo* encourages the celebrants to experience role reversal, pretending to be who they are not. Thus, children or youth masquerade as adults and experiment with adults-only activities such as smoking cigarettes and drinking liquor. Haredi and mainstream secular media have already covered this phenomenon (see, for example, Adamker, 2016; Farkash, 2013). Haredi media validates smoking as normative non-deviant behavior, although this social religious event, in which children pretend to be adults, justifies problematic behaviors which are then afterward adopted. The interviewees told us that since early childhood, they had wanted to smoke as they perceived smoking as a sort of coming of age ceremony.

It seems that the moderate character of the Bar Mitzvah ceremony and its temporality stimulates a yearning to take part in another sort of rite of passage, which is more socially deterministic. Jonathan recalls, "I saw the grown boys smoking and I wanted to do it too. I started on Purim when no one tells you not to do so." His words indicate that during his childhood, he considered this holiday as a chance to achieve his desired self-image. Jonathan briefly mentions the lack of cultural prohibition. In explaining, "No one tells you not to'" Jonathan admits that it enabled him to create a justification system for this holiday that produced an additional ceremonial meaning of maturation.

The interviewees described teens in the Haredi sector as taking pride in the opportunity to experience different kinds of behaviors while enjoying a non-judgmental social attitude that even embraces this custom. The interviewees admitted that after the first cigarette on Purim, they started smoking regularly. Joel described starting in his early childhood:

I started smoking on Purim. When I was a 5-year-old, I already bought a 'Time' [brand] cigarette packet. Every Purim, I smoked three packets. I was permitted to smoke on this occasion.

Beyond Joel's initiation at a very young age, it seems he yearned to validate an absent reactive dimension of his deviant act. Thus, he excessively claims that he used to smoke nearly 60 cigarettes a day. Eli shared that smoking is permitted on every *Yom Tov* ("a good day"), i.e., any Jewish holiday, not only Purim. The halacha forbids lighting fire, yet it does not prohibit a transfer of fire from one flame to another item if the fire is transferred by a candle or otherwise. Thus, Eli was often sent to the neighbor to ask him to light a cigarette for his parents. When he was only 10 years old, he recalls transferring the lit cigarette to his parents *and* smoking it himself, thus quickly adopting this custom. Eli's story is not exceptional. Asaf also used the permission to transfer fire on *Yom Tov* for smoking. Any request to transfer fire was an opportunity to smoke, as he explains:

I grew up in a smokers' house. No particular holiday stimulated smoking. When I had to transfer fire from a window across my parents' house, I took the liberty and started smoking on holidays. It happened when I was little.

In defining his parents' house as "a smokers' house," characterized by frequent smoking by all his family members, Assaf indicates that he did not need to wait to masquerade on Purim. Transferring fire as a young child per his parents' request instigated his perception of smoking as normative, alongside an implied understanding from his parents that there was no option for him but to become an adult smoker. Such consideration is validated by Ira Tolchin from the Israel Cancer Association: "When parents are smoking, and the children see them, it is passed down to the next generation. If there are more smokers in a particular society, then their children are more likely to become smokers. Chances are that a child of smokers will become a smoker himself" (Raban, 2022). Though interviewees describe their anticipation for a holiday to validate their maturity, in narrating their behavior, they did not present it as masquerading adulthood but rather presented an adoption process of smoking as an acceptable norm.

### "And You Shall Safeguard Your Soul"—From Commandment to Recommendation

Researching students in Israel, Koren and Bony-Noach (2011) discovered that numerous students regard the choice to smoke as a health risk, alongside perceiving it as exciting and as a potential psychological release, and some admitted that they smoke when they feel distressed. Likewise, our interviewee, Abraham, perceives smoking as healing:

I know the halachic order "And you shall safeguard your soul." Yet, for some people, smoking is a medicine. If they quit smoking, it will not be good for them. Hence, they do not follow this order.

Though Abraham is fully aware of the halachic rule, mental health precedes physical health in his eyes, and he perceives smoking as no less than a medicine. Other interviewees referred to the contradiction between the said rule, which aims to promote awareness of the importance of maintaining a healthy body, but noted that smoking is exempt, as they perceive smoking as a behavior that helps them to reduce stress. While Abraham regards the halachic rule as a mere statement, Ethan perceives it as an order, yet an order that should not be strictly obeyed. He says:

I know the commandment "And you shall safeguard your soul." Why is smoking on *Yom Tov* permitted? It has been said that it is like food for the soul. A human being needs it for survival. That is why I smoked. I mentally needed to do it. Even when we went abroad, we calculated who smoked and when, so we could smoke and transfer fire from one person to another. This approach suggests that a person won't die if he smokes but, rather, feels better.

Interviewing Ethan became a sort of *Havruta.*^2^ Ethan asks a question and immediately answers it with his interpretation as he negotiates with himself. He suggests that it is allowed to smoke on *Yom Tov* as it helps the human spirit, and it is even an existential need. Ethan attempts to be accurate and exemplifies his attitude with a story about transferring fire on Jewish holidays. He stresses that he and his friends, as they resided abroad, tried to calculate and regulate the transfer of fire from one to another. He finally casts doubt on the risk of mortality, and like Abraham, he considers smoking as a medicine that "will do him good." The skeptical and tolerant approach toward the potential danger has also been demonstrated by other interviewees as well, who consider themselves as part of a risk society (Beck, 1992), feeling that they live in a modern age in which the individual cannot anticipate risks. Thus, smoking, dangerous as it might be, is perceived as one of many other risk factors. Menashe, for example, cites the common belief: "Smoking will not necessarily kill me, as many other things might kill me first." Menashe even claims that smoking is not a health risk, demonstrating his rationalization process:

I think smoking is not dangerous. Statistically, no one dies in his 20s to 40s because of cigarettes. Thus, until he is 60-year-old or 80-year-old, he can die because of so many things. Therefore, cigarettes won't kill me. Many things might kill me, and the cause of my death will not be related to cigarettes. I will die according to God's will. Smoking can be harmful only in the long term. "And you shall safeguard your soul" is more relevant to road safety, e.g., signal when your car turns right or left and, as a pedestrian, look both ways before you cross the street.

Menashe employs the discourse of scientific methodology to explain his presupposition, claiming that "statistically," many factors are involved in mortality, and smoking is not one of them. He also justifies his standpoint by introducing us to the idea of *Bitool Ha'Shalem* ("complete abnegation") in Judaism. This term suggests that God entirely and exclusively controls human fate. Menashe uses the Israeli highway system to exemplify dangerous space, in common with other interviewees. Risky behavior is demonstrated by drivers and their old habits rather than one-time actions. According to this perspective, a person should adopt appropriate behavioral habits over time, such as crossing the road safely and signaling when a car turns right or left since the danger is predominantly immediate. Jacob metaphorizes potential dangers on the roads as well in order to clarify the true meaning of the halachic law:

Smoking is a matter of choice. "And you shall safeguard your soul" relates to many things besides smoking. From my perspective, this commandment has nothing to do with smoking. I believe it is more about dangers on the road and risky things than a human being can do, and it orders one not to risk oneself for no cause.

Jacob explains that associating the risks of smoking with the halachic phrase is misleading. He notes that the phrase relates to a real danger, although it is unclear what he means by adding "not to risk oneself for no cause." Har'el also speaks about dangers on the roads. He describes borderline, suicidal behavior that apparently demonstrates an authentic sense of danger:

I believe that this commandment refers to immediate danger, not to something that can be risky or not. It forbids a pedestrian from jumping onto a highway. I think that most of the Haredi leaders do not deal with it because they think it's a lost battle.

Har'el describes a fairly flexible behavioral axis with two ends. One end is mortality resulting from suicidal behavior ("jumping into a highway"); the other end is a potential yet questionable risk. Then he rationalizes the phenomenon of smoking and suggests that in the Haredi leadership's perspective, prohibiting smoking is unreasonable since it is a decree that the public cannot obey. Rachamim, however, does not regard smoking as a medicine, and he does not metaphorize it. Instead, he tries to deal with the potential contradiction in multilayering the power of halachic commandment:

There is no real definition. You can refer to the idea of "And you shall safeguard your soul" and remember that some *Poskim* [distinguished Halacha interpreters] consider this phrase a strict halachic prohibition. However, it is not a real [prohibition] since it doesn't pass the Halacha test. Nevertheless, it is customary to think that "And you shall safeguard your soul."

Rachamim exposes multilayered prohibitions that oscillate between strict halachic rules, restrictions and explicit commandments while creating a space open to different interpretations. Thus, a Haredi individual might choose not to obey this non-explicit prohibition.

Some interviewees compared smoking to poor nutrition, e.g., consumption of high-cholesterol foods and white sugar. Notably, a healthy diet is commonly perceived by Haredi men as less important than keeping kosher. Evyatar refers to the status of nutrition in Haredi culture, as he considers the smoking prohibition as amorphic in comparison with the determinant kosher laws:

This prohibition is nonexplicit, it cannot be obeyed to the same extent and relevance as the Jewish prohibition of mixing meat and dairy. Associating smoking, which is a bad thing, with the phrase "And you shall safeguard your soul" is inappropriate. One can say that it harms the body, but high-cholesterol food can also be associated with that halachic rule. Speaking about this matter, I would say that if everything relates to "And you shall safeguard your soul," then it would be very problematic on a public level. People who are responsible for their words should never say something unclear.

Evyatar compares a smoking prohibition and explicit laws of religious Jewish dietary requirements laws that coincide with his cultural perception of purity. From a structuralist anthropological perspective, impurity is perceived as threatening and hazardous (Douglas, 2003 [1966]). Smoking, however, is not perceived by Evyatar as a health risk, and he hierarchizes the rabbinic prohibitions and their halachic status. Furthermore, he extensively explains that smoking and the halachic phrase are unrelated since, in his eyes, smoking is a category that is like poor nutrition. Thus, a prohibition against smoking is nothing more than arbitrary medical advice. Comparing smoking with cholesterol coincides with the explanation of Tolchin, who states that smoking is widely perceived as a nonimmediate danger that affects people only decades in the future (Raban, November 2022). By comparing the laws of kashrut and a prohibition against smoking, Evyatar suggests that public responsibility means avoiding imposing a stringent halachic anti-smoking policy. By putting himself in the shoes of the rabbinic authority, Evyatar claims that it is inappropriate to forbid smoking firmly. He considers smoking as slightly risky behavior, which is no more dangerous than poor nutrition, which is usually addressed as part of health recommendations rather than through uncompromising prohibitions.

### Religious Leadership and Smoking as Shared Cultural Behavio***r***

The cultural similarity reflected in similar tastes, experiences, leisure activities, and self-presentation styles shared by members of a particular group (Bourdieu, 1984) is particularly relevant in describing a Haredi man who spends most of his time with his peer group from the time early childhood, as his daily routine is institutionalized. Correspondingly, some interviewees admit that their need to smoke is associated with breaks at the *yeshiva*, sometimes because of boredom.

Smoking in Haredi schools means conformity, as some of the teachers and rabbis smoke in the company of their students. Some of the yeshivas do not seriously consider smoking to be a prohibition. Interviewees described seeing rabbis and even rebbes, distinguished leaders of Haredi Hassidic communities (*Admorim*), who were smokers. Since the Rebbe is a role model for Hassidic society, no wonder smoking became perceived to be no more than a petty sin. The interviewees explicitly talk about the role of a rabbi, as the head of the *Yeshiva*, in providing a personal example. He is expected to embody halachic and healthcare norms by his personal behavior. One of the interviewees reveals that his Rebbe's conflictual behavior, however, arises from the Rebbe's particular relationship with his family members: "The brother of the head of the Yeshiva was a heavy smoker. The Rabbi prohibited smoking before the first class of 11th grade. Eventually, he allowed us to smoke at the dining room and *Beit Midrash.*" It is unclear if the smoking habits of the brother of the head of the Yeshiva were the main reason for the initial smoking prohibition, and it seems that the prohibition was temporal. The interviewees revealed no consistent prohibition or standard in Haredi educational institutions. For example, Pinhas says:

According to Halacha, smoking is life-threatening. No Rabbi said that a smoker might die because of this habit, yet Rabbis regularly preach, "And you shall safeguard your soul." Smoking is prohibited in *Yeshiva Ketana*^3^ and permitted in *Yeshiva Gedolah*.^4^ It is so today, though e-cigarettes smokers at *Yeshiva Ketana*, too. The students smoke discreetly, not openly, because they do not wish to be temporarily or permanently suspended.

At first, Pinhas notes that the Haredi education system, inspired by the halacha, acknowledges the mortality risk. Yet, he adds, not even one rabbi directly associated cigarettes with death beyond the formal halachic commandment. Going further, he recognizes a sort of hierarchized surveillance in which the supervision of small *yeshivas* enforces smoking prohibition but then withdraws. Some interviewees related to an existing prohibition that was not enforced: "In many yeshivas smoking is prohibited, but in numerous others, it's allowed, mainly in Lithuanian yeshivas. Also, in big yeshivas, it's officially prohibited but unenforced, similar to the one I attended." It seems that the official position is openly negating smoking, but implicitly, the prohibition is not enforced. In response to our question, the interviewees replied that they are unfamiliar with a rabbi who forbids smoking, including the rabbi of their own congregation. They explain that even if the rabbi disagrees with this custom, he never preaches against it. Furthermore, some interviewees explicitly say that if the rabbi of their congregation bans smoking, they will still keep smoking anyway; as Yosef explains: "The congregation’s rabbi didn’t say anything against smoking. His position is negative. Rabbis who are smokers will find a reason to keep smoking, like the distinguished Rabbi Yitzhak Kaduri, who smoked until he was over 90-year-old, and he was fine." Yosef puts himself in the shoes of rabbis who are smokers, and he assumes that they would "find a reason" for justifying their smoking habits. Further, he rationalizes his attitude by not perceiving smoking as health-risky, and he exemplifies it by referring to the distinguished and famous Rabbi Kaduri, who kept smoking even at a ripe old age.

Some interviewees claimed that although distinguished halachic interpreters have "written" that smoking is forbidden, their interpretation is nonbinding as opposed to a halachic ruling that should be obeyed. Yitzhak explains:

Intricate standpoint. Distinguished Haredi interpreters have recently prohibited smoking, and great rabbis have in recent decades. The official stand is against smoking regarding "and you shall safeguard your soul." Yet this is not exactly a halachic prohibition; people didn't really accept it and kept smoking everywhere.

Being mindful of the Haredi interpreters' standpoint, Itzhak perceives it as an intricate issue. The rabbis’ attitude is not perceived as a strict prohibition, so his stance is double-sided. Reuven also speaks about disobedience. In putting himself in the shoes of his rabbis, he acknowledges the potential threat to the rabbinic authority embedded in overruling: "No rabbi deals with smoking prohibition thing. Sometimes people can find themselves not obeying, which is worse." In titling the prohibition as a "thing," Reuven undervalues it as a passing trend. His statement indicates that smoking is a conforming act and addresses a fundamental psychological need. Thus, its prohibition might stimulate a rebellion. A threat hovers over the head of a rabbi who bans smoking since disobeying rabbinical authority *is* the real danger.

Advancing health among Orthodox and Haredi communities necessitates the active involvement of their rabbinical authorities (Flannelly et al. 2006). Conspicuously, smoking is perceived as a norm by Haredi society, as demonstrated by the rabbis' efforts to promote kosher cigarettes for Passover. In 2013, distinguished halachic interpreters deliberated on this issue, and, eventually, an Israeli cigarette company allowed Haredi kashrut supervisors to enter its factories and confirm that the local cigarettes are kosher for Passover. For years, cigarette companies refused to expose the cigarettes' ingredients and odor stabilizers, which meant that a reliable halachic standpoint was not determined. Consequently, part of the Haredi public avoided smoking on Passover and its intermediate days. This issue was solved, though, and the local cigarettes were certified as kosher for Passover (Nachshoni, 2013). Notably, some interviewees underestimated the efforts to make cigarettes kosher for Passover and implicitly criticized them; as Shimon says: "There used to be cigarettes kosher for Passover like they said that Tobacco contains *not* kosher ingredients for Passover!? No one bought kosher for Passover cigarettes." A critical perspective on making kosher cigarettes seems to stimulate Haredi customers to avoid kosher cigarettes. In relating to a potential *risk of impurity* (Douglas, 2003 [1006]), Benjamin says:

On Passover, almost all the rabbis opposed smoking due to the fear of existent non-kosher ingredients in the cigarettes, and yet many Haredim keep smoking non-kosher cigarettes. Their addiction overpowers them. As long as smoking is not life-threatening or as bad as eating pork or such offense, it won't be prohibited. Possibly, rabbis realized that such a prohibition would be a decree that the public could not observe; thus, they did not ban smoking. There is no total smoking ban.

In perceiving smoking as addictive, Benjamin explains how physical addiction overpowers halacha. He compares smoking to eating kosher and demonstrates how non-kosher food is perceived as life-threatening and endangering (Douglas, 2003 [1966]). In his comparison, eating pork is life-threatening, as it represents complete impurity. As smoking is perceived as less offensive, there is no room for a total ban.

The interviewees related to differences in smoking habits between various Haredi sectors. Hassidic communities demonstrate a more forgiving attitude than Lithuanian and Mizrahi communities. Some explained that smoking is considered by Hassidic communities as an accepted norm, in contrast to Zionist communities. The interviewees thoroughly described the Ger Hassidic Sect, in which smokers are a minority since this is the only Hassidic sect that is led by a Rebbe who strictly prohibits smoking. Yaacov Litzman, formerly Minister of Health and a member of the Ger Hassidic Sect, indicates, "Even if someone celebrates his engagement, his guests enjoy chocolates, no cigarettes" (Grodka, 2017). Litzman’s words demonstrate a norm in Haredi society to offer cigarettes to guests at festive events, which is probably inspired by the American custom of offering celebratory cigars (Schwartz, 1967). The interviewees emphasized the severe attitude of the Ger Hassidic Sect, which focuses on taking care of the community's health, as they related a news item about the Rebbe of Gur, who gave his blessing to members of his congregation who were leaving Haifa due to a published report about hazardous air pollution in that city. This news item, however, relates to the Rebbe's blessing for members of his sect who left Tel Aviv due to its increasing secularity (Shaul, 2020).

Interviewees analyzed the concept of a prohibition against smoking in other religious frameworks as well, and they connected the potential successful prohibition to the social positioning of the rabbi:

Haredi public can say, "And you shall safeguard your soul," but I think it's the least careful public; they don’t cross the street on a crosswalk, they eat unhealthy food and reside in unsafe buildings. Also, the Haredi public cares a lot about spirituality and obeying the commandments, yet it doesn't care much about materiality. Rabbis that related to "And you shall safeguard your soul" are not part of the mainstream. Rabbi Yechezkel Ishayek, for example, is not one of the distinguished halacha interpreters despite his proximity to the distinguished Rebbe Schach.

Asher explains, in the plural, as representing the Haredi public and divides his claim to material logic as opposed to spiritual. He criticizes the lack of careful behavior in the Haredi sector, yet he explains that this recklessness is only about the bodily world. Though mindful of the biblical phrase, they engage in various hazardous behaviors. Asher emphasizes the Haredi spiritual perception. Going further, Asher relates to Rabbi Ishayek's book *Life without Smoking According to the Torah* (self-published, 2003). In his attempts to convince his readers to quit smoking, Ishayek details the crucial need to protect health according to the Torah and the halacha, explains the risks entailed and validates an anti-smoking approach by the rules of halacha. Asher's words bring to mind the Mayor of Jerusalem’s 2008 campaign, in association with other mayors and the Haredi leadership, attempting to abolish smoking within the Haredi community. This campaign was unsuccessful due to the absence of influential rebbes' statements against smoking. In his hierarchization of rebbes, Asher undervalues Rabbi Ishayek's status. He contends that despite Rabbi Ishayek's higher position as one of the leaders of the Shas party of Sephardic Haredim—and his previous role as an assistant of the distinguished Rebbe Eliezer Menachem Schack, the former head of the Ponevezh *Yeshiva*—Rabbi Ishayek is not regarded by the Haredi community as one of the most distinguished interpreters of halacha. Similarly, Sholem, a Lithuanian Haredi yeshiva student, relates to the social positioning of rabbis as potentially able to generate change:

If I noticed a clear standpoint of one of the *Gedoylim*^5^ that considers smoking as *Isur Hamur* [strictly prohibited], as a halachic offense, then I would stop smoking immediately. I would not be smoking! But no, there's no such thing! I don't know of such a thing. Obviously, it's unhealthy, but it's not a big disaster either. If there was an absolute prohibition you would hear it from Rebbe Elyashiv, Rebbe Kanievsky, Rebbe Steinman, Rebbe Auerbach, or the head of a yeshiva. Can you show me that any of them categorize smoking as *Isur*?!

Sholem's words exemplify the intricate logic that typifies his social group. First, he considers "prohibition" as an absolute category that must be obeyed, implying that a strict prohibition means a halachic offense, *Isur Hamur* in Hebrew, that should not be ignored. In such a case, he would clearly obey. His further explanation suggests that categorizing smoking as *Isur* can only be carried out successfully by a great interpreter of halacha. The *Gedoylim* are well-known, credentialed rebbes perceived by Haredi society, particularly the Lithuanian Haredim, as an institution in their own right and as ultimate power sources. Since these rebbes have never manifested a "clear standpoint" as Sholem puts it, smoking is perceived as a minor risk. From this perspective, the Israeli Ministry of Health's recommendations are merely arbitrary, in contrast to "real prohibition" which depends on a rabbinic prohibitor. Sholem specifies names of leaders of the Haredi Lithuanian public in the recent decade, and he contends that only a prohibition made by these rebbes should be unquestionably obeyed.

## Discussion: Changing the Status of a Permissible Sin

Our findings indicate that in accordance with the Israeli Ministry of Health's report (IMH, 2011), most of the interviewees in our research do not wish to quit smoking, although they are mindful of various halachic interpretations that prohibit smoking. Research indicating low rates of tobacco cravings and nervousness on Saturdays, in comparison with weekdays (Dar et al., 2005), also demonstrates the unique characteristics of this sector, alongside potentially explaining the vast difference between the interviewees' attitude toward not smoking and obeying kashrut laws. As shown in Evyatar's assertion in the findings, it is clear that not adhering to Kashrut laws coincides with concepts of purity, whereas smoking does not.

There is also a need to acknowledge the power of one's self-persuasion in motivating the fulfillment of a subjective will and consequent production justifications aimed at validating one’s choice (Winchester & Green, 2019). Thus, some of the interviewees demonstrate a skeptical approach toward the health risk aspects of smoking, which corresponds with Petek et al. (2006) findings. However, it is important to emphasize that individuals within the Haredi community can be potential agents of change, as reflected in the interviewees' description of a refusal to buy kosher for Passover cigarettes—as Shimon and Benjamin dismissively express in the findings. These individuals demonstrate a critical standpoint, describing their acts as carefully planned. Hence, it is essential to make efforts to refute false information and to provide reliable information about the dangers of smoking and, particularly, refuting the failed presupposition that smoking is a long-term stress reliever (Silverstein, 1982; Kessel et al., 2003).

The health authorities' actions against smoking in the Haredi sector need a careful cultural adjustment (Taragin-Zeller & Stadler, 2022; Taragin-Zeller et al., 2022). Following Anshel's Disconnected Values Model (DVM) (2010), which promotes intervention by religious leaders to change their congregation's lifestyle, it is worth emphasizing the importance of a unified message from the Haredi leadership. This was also expressed by Sholem, who stressed that rabbis acted to irradicate smoking were not one of the Gedoylim; thus, no association to *Isur Hamur* was made. Accordingly, values and ideals should initially be cultivated through *Isur*, i.e., a strict halachic prohibition against smoking. Such a prohibition declared by the Haredi leadership will stimulate anti-smoking actions, especially since the scholarship indicates the massive power of religious organizations in advancing health issues among their members (Flannelly et al. 2006; Schoenberg, 2017). As actions to eradicate smoking were previously taken, e.g., the 2008 campaign, it is worth drawing a lesson and advancing the intervention of influential rabbinic leaders. As our interviewees suggested, such intervention can potentially resolve the problem of poor enforcement in Haredi educational institutions.

The interviewees demonstrated diverse rationalization processes in regard to the power of the commandment "And you shall safeguard your soul" and its status. Some, like Yosef and Ev'yatar, put themselves in the shoes of the Haredi leadership by stressing that the rabbis themselves do not believe it. Yet our findings indicate that despite a significant desire to follow this risky custom, the power of normative prohibitions within Haredi society can be instrumentalized to prohibit smoking. Equating the status of a halachic smoking ban to the status of kashrut laws, i.e., *Isur Hamur (*strict halachic prohibition), will generate a change of mind, as we learned from the interviewees' attitudes, as Sholem passionately expressed, that if smoking will be strictly prohibited "I would stop smoking immediately. I would not be smoking!". The importance of strict halachic prohibition is deeply implemented in their culture; the fact that smoking does not have such a prohibition means that it is misperceived as a minor risk.

The interviewees' description of an existing prohibition of lighting fire while permitting a transference of fire on a *Yom Tov,* exemplifies parental enabling of adopting a risky behavior, as Eli shared from his childhood experience. Hence, an integral part of the construction of norms regarding the abolition of smoking in Haredi society should be directed to appropriate parental guidance, highlighting the importance of a personal example. Additionally, ethical advocacy of a prohibition against smoking that emphasizes religious values and faiths can be advanced through personal examples among other circles of belonging, including heads of yeshivas and rabbis. Further, according to Rizier's recommendation (2017), religious thinking should be considered in preventive medicine actions, and traditional religious perceptions should be employed to advance public health.

Cultural adjustment for an intervention program to eradicate smoking should not obligate extensive professional training for a community rabbi, nor should he be a certified psychologist. Instead, it should highlight the need to emphasize the existent contradiction between adopting a dangerous behavior and the old Sages' commandment "And you shall safeguard your soul." Emphasizing this contradiction not only highlights the negative consequences of smoking but also presents smoking as disconnected from values and faiths.

Moreover, it is important to challenge the perception of smoking as normative conduct on Purim and festivals. As mentioned above, our findings indicate that the young Haredi man starts smoking at an unusually young age, and he even regards smoking as a sort of coming of age ceremony that advances his self-image as an adult smoker. Yet Haredi society does not accept the idea that smoking is a normative deviance and blocks the possibility of considering smoking as reactive deviance that relies on an exterior critical perspective (Goode, 2019). Thus, smoking is not perceived as a reactive deviance on Purim, as was exemplified by Jonathan and Joel in the findings, on which many Haredi children experiment with smoking as part of the custom of *venahafoch hu (*turning everything upside down).

Pinchas-Mizrachi and Finkelstein (2022) emphasized the connection between socioeconomic status and religiosity level, thus indicating a need to tailor the intervention program (Pinchas-Mizrachi & Finkelstein, 2022). Also, following the recommendations of Knishkowy et al. (2012) to prohibit smoking in educational institutions and take measures to de-normalize the smoking phenomenon within the community—in our conclusions, we illustrate paths toward intervention that will emphasize the cultural aspect in order to future tailor an operational program.

## Conclusions: Illustrating Paths Toward Intervention

**Fig. 1:**
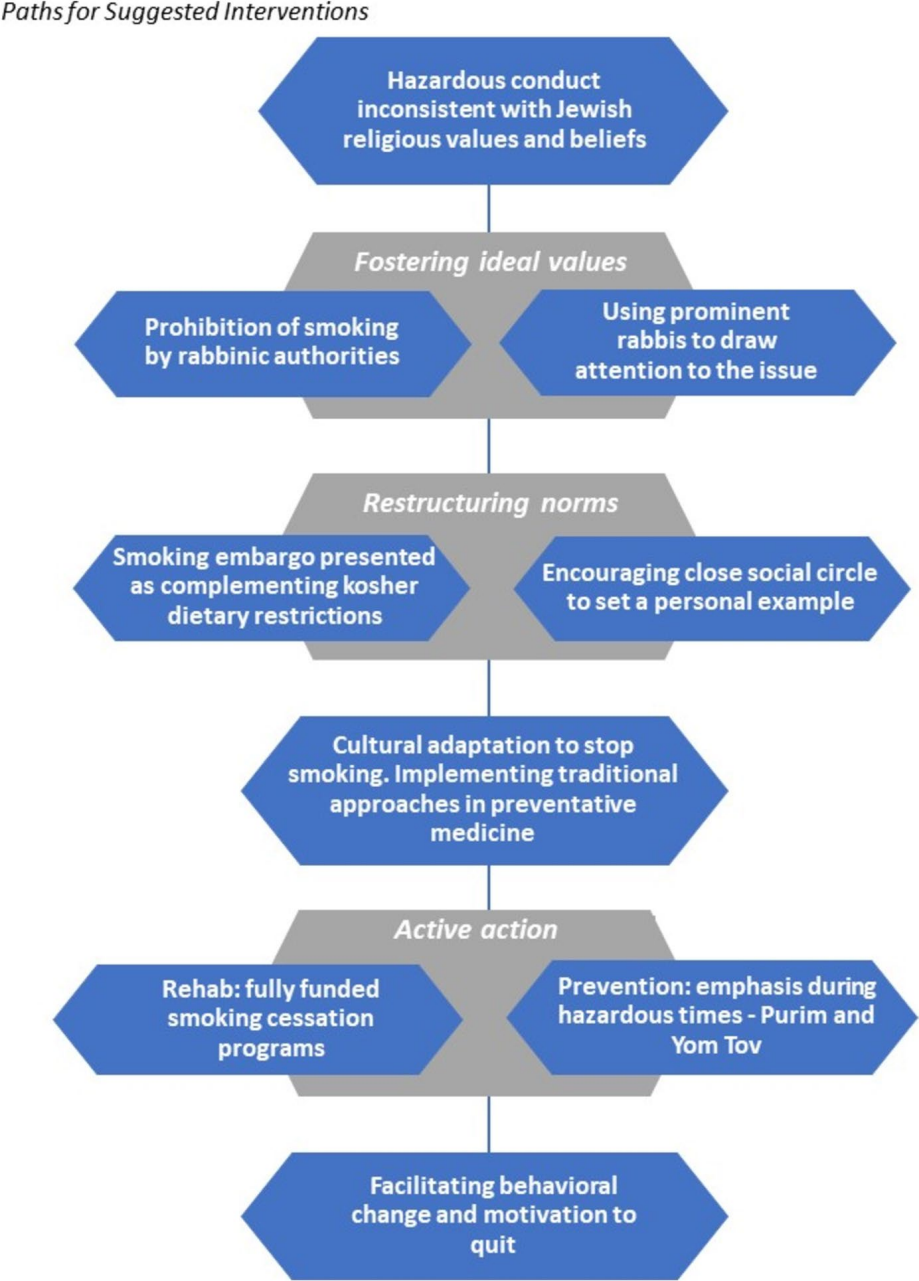
Paths toward interventions

In Fig. 1, we present paths leading to intervention. The illustration below facilitates a better understanding of culturally tailored intervention programs for the abolition of smoking within the Haredi population in Israel.

The paths illustrated to facilitate intervention aimed to generate a behavioral change and a mindset shift, which is essential for quitting smoking. The abolition of smoking in Haredi society needs institutionalized governmental cooperation with the halachic Haredi establishment to encourage taking proper action. Notably, the Haredi public is characterized by its separate education, consumption, and leisure systems. Accordingly, motivating smoking cessation should involve designated and culturally tailored programs for this public while offering tobacco cessation programs subsidized by the state.

The limitations of this research are twofold: The first relates to the broad spectrum of ages and diverse subgroups within the Haredi sector, revealing that smoking is a prevalent behavior. However, this also posed limits to the research in terms of obtaining specific replies. Our research findings are qualitative; as such, they assist in understanding a phenomenon and the participants' experiences. Thus, future research (probably quantitative with a large sample) will be able to substantiate data toward the operative model.

In conclusion, a combination of enlisting the rabbinical establishment, initiating large-scale designated information campaigns, and community-level actions will be very helpful in establishing a prohibition against smoking that is stricter and more meaningful in terms of being a *religious* prohibition than is the case today. In addition, the state should specifically intervene by financing designated programs for smoking cessation among the Haredi public, with particular attention to holidays like Purim on which many children and youngsters start smoking. A reasonable combination of these ingredients has great potential for creating a holistic, comprehensive intervention program that successfully deals with the problematic perceptions of smoking among many Haredi men. Comprehensive and culturally adjusted action may advance a perceptual change and, consequently, a behavioral change among the Haredi public concerning smoking or, at least, it might result in a change that disrupts the typical starting point of smoking and stimulates both public and individual motivations to quit smoking.

## Footnotes

1 In 2020, they comprised 12.6 percent of the Israeli population (Malach & Cahaner, 2021).

2 A fellowship—traditional Jewish group learning of a shared text.

3 Small *Yeshiva*, a Haredi elementary school and middle school.

4 Big *Yeshiva*, a Haredi educational institution for secondary and post-secondary students.

5 A prominent Rebbes who are considered as great Halacha interpreters.
